# Inactivation of *Fam20B* in Joint Cartilage Leads to Chondrosarcoma and Postnatal Ossification Defects

**DOI:** 10.1038/srep29814

**Published:** 2016-07-13

**Authors:** Pan Ma, Wenjuan Yan, Ye Tian, Jingya Wang, Jian Q. Feng, Chunlin Qin, Yi-Shing Lisa Cheng, Xiaofang Wang

**Affiliations:** 1 Department of Biomedical Sciences and Center for Craniofacial Research and Diagnosis, Texas A&M University Baylor College of Dentistry, Dallas, Texas, United States of America; 2 Department of Oral Implantology, Beijing Stomatological Hospital, Capital Medical University, Beijing, People’s Republic of China; 3 Department of Diagnostic Sciences, Texas A&M University Baylor College of Dentistry, 3302 Gaston Ave, Dallas, TX, United States of America

## Abstract

During endochondral ossification, chondrocytes embed themselves in a proteoglycan-rich matrix during the proliferation-maturation transition. Accumulating evidence shows that proteoglycans are essential components for chondrocyte proliferation and differentiation. When we conditionally inactivated *FAM20B* (Family with sequence similarity 20 member-B), which is a newly identified xylose kinase essential for glycosaminoglycan (GAG) formation on the protein core of proteoglycans, from the dental mesenchyme using *Osr2-Cre*, which is also strongly expressed in joint cartilage, we found chondrosarcoma in the knee joint and remarkable defects of postnatal ossification in the long bones. Mechanistic analysis revealed that the defects were associated with gain of function in multiple signaling pathways in the epiphyseal chondrocytes, such as those derived by WNT, BMP, and PTHrP/IHH molecules, suggesting that the FAM20B-catalyzed proteoglycans are critical mediators for a signaling balance in the regulatory network controlling chondrocyte differentiation and proliferation. In particular, we demonstrated that the WNT inhibitor was able to rescue part of the bone defects in *Osr2-Cre;Fam20B*^*fl/fl*^ mice, indicating that *FAM20B*-catalyzed proteoglycans regulate postnatal endochondral ossification partially through the mediation of WNT signaling.

In vertebrate animals, most bones develop through a process known as “endochondral ossification”, during which the osteoblasts and bone matrices gradually encroach upon the chondrocytes and cartilagionous matrix after the establishment of primary and secondary ossification centers at the middle and each end of the cartilage templates^1^. The postnatal chondrocyte differentiation and bone elongation are under tight control by multiple signaling pathways that form a signaling balance between the growth plates and periarticular cartilage, such as those directed by WNTs, bone morphogenetic proteins (BMPs), fibroblast growth factors (FGFs), Indian hedgehog (IHH), and parathyroid hormone-related peptide (PTHrP), which are required for the proliferation and differentiation of chondrocytes^2^,^3^. During chondrocyte differentiation, WNT signaling is generally believed to promote chondrocyte hypertrophy and final maturation^4^,^5^, BMPs induce chondrocyte proliferation and maturation^6^, while PTHrP and IHH are known to mediate chondrocyte hypertrophy by forming a negative feedback loop^7^.

Compared to these relatively well understood morphogens and growth factors that mediate chondrocyte differentiation, the biological function of proteoglycans that constantly interact with these secreted signaling molecules on the chondrocyte surface and in the extracellular matrix remains poorly understood. During endochondral ossification, the chondrocytes embed themselves in a proteoglycan-rich matrix. Glycosaminoglycan (GAG) chains covalently attached to the core proteins of proteoglycans provide docking sites for signaling molecules and their receptors^8^. Accumulating evidence shows that proteoglycans orchestrate signaling pathways by mediating cell-cell and cell-matrix signaling and help control gradient diffusion of morphogens and growth factors^9^,^10^. Numerous studies have demonstrated that disruption of the GAG assembly or proteoglycan core proteins causes defects in cartilage and bones^11^.

Family with sequence similarity 20 (FAM20) is a group of evolutionarily conserved molecules comprised of FAM20A, FAM20B and FAM20C. FAM20C is a Golgi-enriched kinase phosphorylating a wide spectrum of secretory proteins including the “small-integrin-binding ligand, N-linked glycoproteins” (SIBLINGs) that are critical for bone formation^12^,^13^. *Fam20C*-knockout mice develop severe hypophosphatemic rickets due to an increased renal phosphate wasting that is likely attributed to the remarkable elevation of serum FGF23^14^. FAM20A is believed to be a pseudo-kinase that forms a functional complex with FAM20C to enhance extracellular protein phosphorylation^15^. Loss-of-function of FAM20A leads to enamel renal syndrome^16^ without significant disturbance in bone formation. FAM20B is a xylose kinase essential for glycosaminoglycan (GAG) assembly^17^. Loss-of-function mutations in *Fam20B* of zebra fish cause extensive cartilage and skeleton defects^18^, while constitutive inactivation of *Fam20B* in mice leads to embryonic lethality at E13.5^19^.

In a recent study, we generated a *Fam20B*-flox allele in mice and inactivated *Fam20B* in the epithelial tissues (including the dental epithelium), which led to supernumerary incisors and enamel defects^20^. In this study, we crossbred the *Fam20B*^*flox/flox*^ mice with *Osr2*-Cre transgenic mice^21^ to inactivate *Fam20B* in the epiphyseal cartilage. The *Osr2-Cre;Fam20B*^*flox/flox*^ mice showed chondrosarcoma in the knee joint and remarkable defects of postnatal ossification in the long bones.

## Results

### The *Osr2-Cre;Fam20B* ^*flox/flox*^ mice showed elongation and ossification defects in long bones

**Figure 1:**
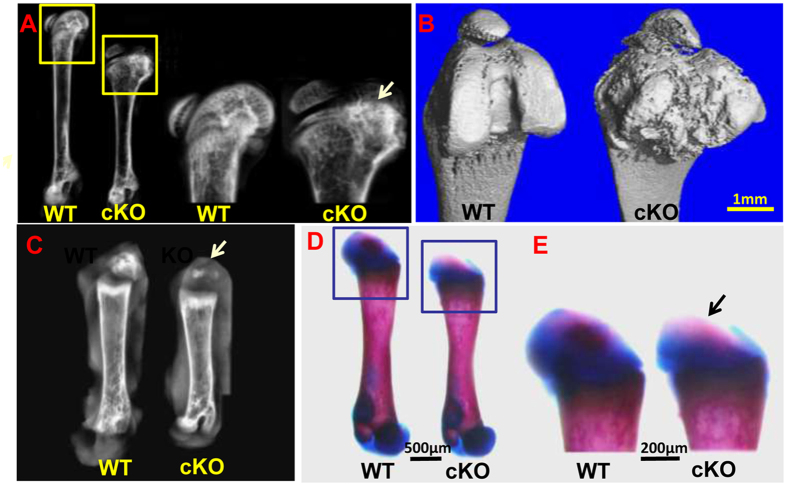
The *Osr2-Cre;Fam20B*^*flox/flox*^ mice showed defects in the secondary ossification centers and postnatal bone elongation. (**A)** Plain X-ray radiography of 30-day-old (P30) mice showed shorter length and wider metaphysis of the femurs from *Osr2-Cre;Fam20B*^*flox/flox*^ (cKO) mice compared with the wild type (WT) mice. (**B)** Micro-CT showed malformed metaphysis and overgrown protrusions on the joint surface of the cKO mice compared with the WT mice. (**C)** Plain X-ray of P7 mice showed a smaller secondary ossification center (arrow) in cKO mice than that in the WT mice. (**D)** The femurs of cKO mice were shorter than that in the WT mice. (**E)** The secondary ossification center of the cKO mice (arrow) showed smaller size and lighter staining of Alizarin red (indicating a lower level or smaller area of ossification) than those in the WT mice. N = 6 per group. Scale bars, 1 mm in **B**, 500 μm in **D**, 200 μm in **E**.

**Figure 2:**
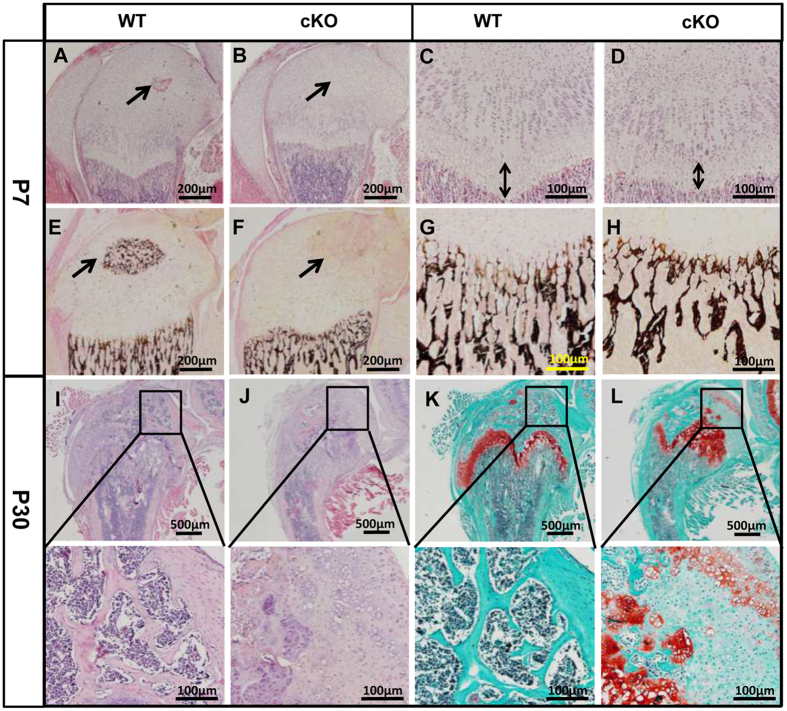
Endochondral ossification defects in the *Osr2-Cre;Fam20B*^*flox/flox*^ mice. (**A**,**B**) H&E staining of the femurs from 7-day-old (P7) mice showed delayed vascularization (arrow) in the secondary ossification center of cKO mice. (**C**,**D**) The cKO mice had a slightly narrower growth plate (double-headed arrows) than that in the WT mice. (**E**,**F**) *Von Kossa* staining of the femurs from P7 mice showed delayed mineralization of the secondary ossification center in the cKO mice. (**G**,**H**) *Von Kossa* staining showed less trabecular bone formation under the growth plate in the cKO mice. (**I**,**J**) The cKO mice had poorly differentiated chondrocytes at the popliteal side of joint compared to normal mice. (**K**,**L**) Safranin O staining showed undifferentiated epiphyseal cartilage and malformed growth plates at the popliteal side of the epiphysis in the cKO mice. N = 6 per group. Scale bars, 200 μm in **A, B, E** and **F**. 100 μm in **C, D, G, H,** and the lower panels of **I–L**. 500 μm in the upper panels of **I–L**.

The *Osr2-Cre;Fam20B*^*flox/flox*^ (cKO) mice do not show apparent defects in the first 5–6 days after birth. At postnatal 7 days, they start to show the defects of bone elongation and overgrowth of joint cartilage (Fig. 1). Plain X-ray of 30-day-old (P30) cKO mice revealed a shorter length and a wider metaphysis in the femurs compared with wild type (WT) (Fig. 1A). The joint of cKO mice showed a malformed shape and overgrown protrusions on the surface in contrast with the normal shape and smooth surface in WT mice (Fig. 1B). The femurs of P7 cKO mice had a smaller secondary ossification center (Fig. 1C) and a shorter length (Fig. 1D), as well as a lighter staining of Alizarin red in the secondary ossification center compared with the WT (Fig. 1E), indicating that the cKO mice had ossification and elongation defects in long bones. The onset of ossification in the secondary ossification centers was remarkably delayed in the cKO mice (Fig. 2). In particular, the epiphyseal chondrocytes located at the popliteal side of the joint in cKO mice were not transformed into spongy bones, while those at the patella side were changed (Fig. 2). The cKO mice did not show vascularization in the secondary ossification center at P7 stage as did in the WT mice (Fig. 2A,B), and their growth plates were narrower than the WT (Fig. 2C,D). *Von Kossa* staining clearly showed a delayed mineralization in the secondary ossification center (Fig. 2E,F) and less trabecular bone formation under the growth plate in the cKO mice. Interestingly, the cKO mice had spongy bone formation at the patella side of the epiphysis, whereas the popliteal side was composed of poorly differentiated chondrocytes (Fig. 2G,H). This pattern is consistent with that of the Safranin O staining (Fig. 2I,J) and the expression pattern of *Osr2*-Cre (see below).

### GAG deficiency in the *Fam20B*-deficient chondrocytes

**Figure 3:**
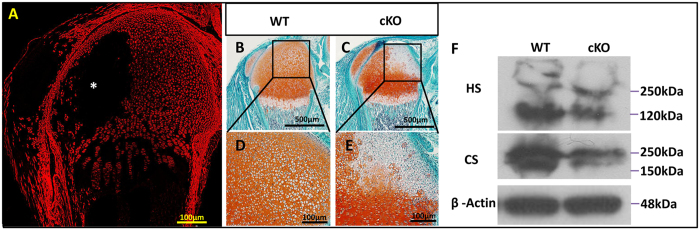
The *Osr2*-Cre and GAG-deficiency demonstrated a comparable pattern in the epiphyseal cartilage of *Osr2-Cre;Fam20B*^*flox/flox*^ mice. (**A**) A sagittal section of femurs from P4 *Osr2-Cre;R26-Tomato* mice showd a strong expression of *Osr2*-Cre in the periarticular cartilage and the popliteal side of epiphyseal cartilage, while the patella side of the epiphysis largely had no Cre expression (asterisk). (**B**,**C**) Safranin O staining on sagittal sections of femurs from P4 mice showed remarkably less staining at the popliteal side of epiphyseal cartilage in the cKO mice, which was comparable to the *Osr2*-Cre expression pattern in **A**. (**D**,**E**) Higher magnification of the boxed areas in **B** and **C**. (**F**) Western immunoblotting showed a reduction of HS and CS in the epiphyseal cartilage of the cKO mice compared with those in the WT littermates. Gels have been run under the same experimental conditions. The blots of HS and CS were cropped from full-length gel as indicated in Figure S A and B. Scale bars, 100 μm in **A**; 500 μm in **B** and **C**, 100 μm in **D** and **E**. N = 6 per group.

Using Tomato fluorescence as an indicator, we observed a strong expression of *Osr2*-Cre in periarticular cartilage and the popliteal side of epiphyseal cartilage, as well as a sporadic expression in the growth plates (Fig. 3A). The location of GAG-deficiency, which was indicated by a remarkably reduced Safranin O staining (Fig. 3B–E), exhibited a comparable pattern to that of *Osr2*-Cre expression (indicated by Tomato fluorescence) (Fig. 3A). Western immunoblotting analyses showed reduced amounts of heparan sulfates (HS) and chondroitin sulfates (CS) in the epiphyseal cartilage of cKO mice compared with the WT mice (Fig. 3F).

### Overproliferation and underdifferentiation of the *Fam20B*-deficient chondrocytes

**Figure 4:**
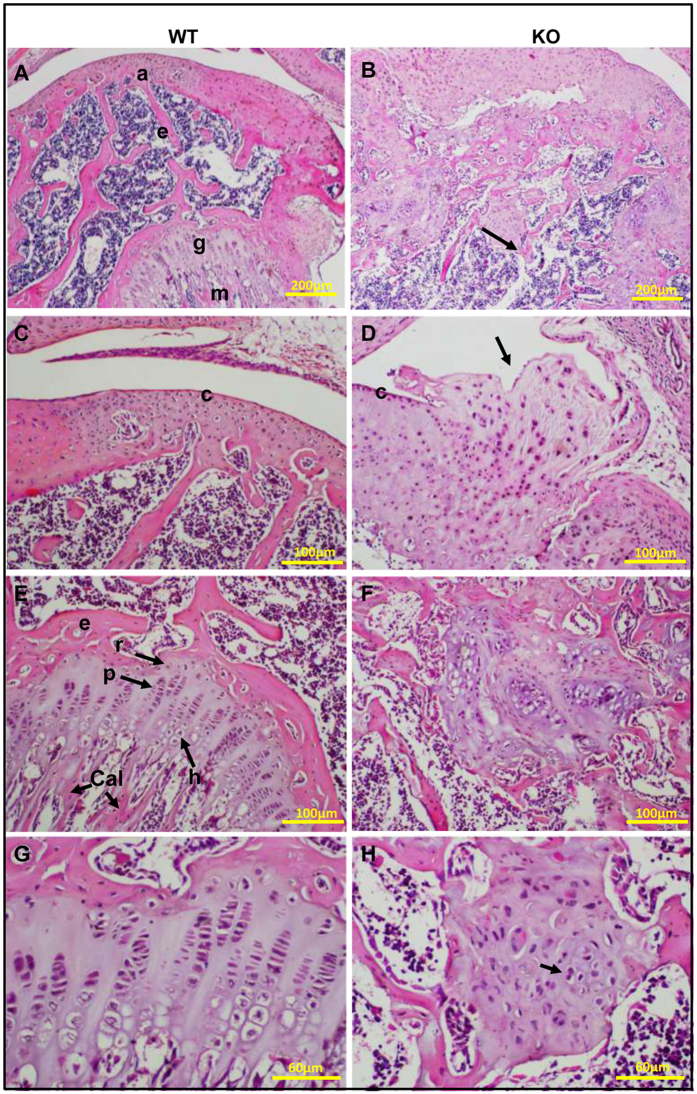
Chondrosarcoma in the knee joint of 7-week-old *Osr2-Cre;Fam20B*^*flox/flox*^ mice. (**A**,**B**) H&E staining on sagittal sections of femurs from 7-week-old mice showed well-developed articular cartilage (a), epiphysis (e), growth plate (g) and metaphysis (m) in the WT. However, abnormal growth and deformity of the femur condyle and disruption of the growth plate are noted in the cKO mice (arrow). (**C**,**D**) The chondrosarcoma perforated the circumferential zone (c) and the articular surface, and invaded the synovial space (arrow). (**E**,**F**) The neoplastic cells in cKO showed chondroid differentiation, and lacked the normal maturation pattern of the hyaline cartilage of the growth plate (r: reserve zone; p: proliferating zone; h: hypertrophic zone; Cal: zone of provisional calcification). (**G**,**H**) The neoplastic cells showed hyperchromatic nuclei, binucleation (arrow), and pleomorphism. N = 6 per group. Scale bars, 200 μm in **A** and **B**, 100 μm in **C–F**, 60 μm in **G** and **H**.

The epiphyseal cartilage of the *Osr2-Cre;Fam20B*^*flox/flox*^ (cKO) mice showed abnormal growth and deformity in the femur condyle and disruption in the growth plates (Fig. 4A,B). The overgrown chondrocytes perforated the circumferential zone and the articular surface, and protruded to invade the synovial space (Fig. 4C,D), the epiphyseal bone and marrow spaces (Fig. 4E,F). The neoplastic cells in cKO mice showed chondroid differentiation, and the normal maturation pattern of the hyaline cartilage of the growth plate was not seen (Fig. 4E,F). The neoplastic cells showed cellular atypia such as hyperchromatic nuclei, binucleation, and pleomorphism (variations in size and shape) (Fig. 4G,H). These battery of pathology are consistent with a diagnosis of chondrosarcoma.

**Figure 5:**
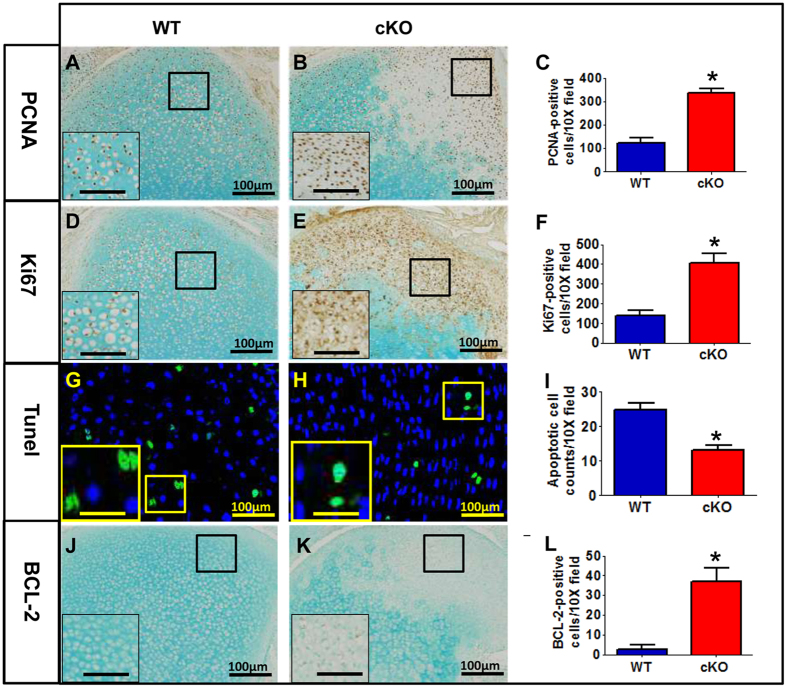
Overproliferation and less apoptosis of epiphyseal chondrocytes in the *Osr2-Cre; Fam20B*^*flox/flox*^ mice. (**A–C**) IHC staining of PCNA showed more expression in the epiphyseal cartilage of P4 cKO mice than that in the WT mice. (**D–F**) IHC staining of Ki67 showed increased expression in the epiphyseal cartilage of the cKO mice. (**G–I**) TUNEL staining revealed fewer apoptotic chondrocytes in the epiphyseal cartilage of cKO mice than those in the WT mice. (**J–L**) IHC staining of BCL-2 showed upregualtion in the epiphyseal cartilage of the cKO mice. A higher magnification (Scale bars, 33 μm) of the boxed areas is shown at the left lower corner in each panel. The average number of positively stained cells was calculated based on the counting of 10× microscopic fields (n = 6) and expressed as mean ± SD. *p < 0.01. N = 6 per group. A Student’s t-test was used to compare the means between two groups. Scale bars, 100 μm.

The chondrosarcoma-like chondrocytes showed overproliferation and less apoptosis. IHC staining of proliferating cell nuclear antigen (PCNA) and Ki67 showed a remarkable increase in the epiphyseal cartilage of P4 cKO mice (Fig. 5A–F). TUNEL staining demonstrated fewer apoptotic chondrocytes in the epiphyseal cartilage of cKO mice than those in the WT mice (Fig. 5G–I). Accordingly, B-cell lymphoma 2 (Bcl-2), an anti-apoptotic protein, was upregualted in the epiphyseal cartilage of the cKO mice (Fig. 5J–L).

**Figure 6:**
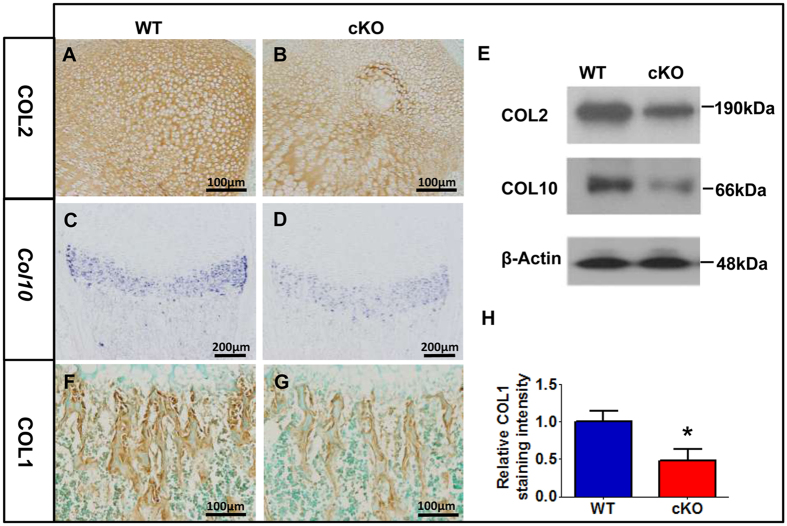
The chondrocyte differentiation defects in the *Osr2-Cre;Fam20B*^*flox/flox*^ mice. (**A**,**B**) IHC staining showed a downregulation of COL2 in the epiphyseal cartilage of femurs in the P4 cKO mice. (**C**,**D**) ISH showed less *COL10* mRNA in the growth plate of cKO mice compared with that in the WT. (**E**) Western immunoblotting confirmed the downregualtion of COL2 and COL10 in the cKO mice. Gels have been run under the same experimental conditions. The blots of COL2 and COL10 were cropped from full-length gel as indicated in Fig. SC and D. (**F–H**) IHC staining showed less COL1 in the trabecular bone of femurs in the P4 cKO mice than normal. *p < 0.01. N = 6 per group. A Student’s t-test was used to compare the means between two groups.

In addition to the overproliferation, the *Fam20B*-deficient chondrocytes also showed underdifferentiation. Type II (COL2) and Type X collagen (*COL10*) were downregulated in the epiphyseal cartilage (Fig. 6A,B,E) and the growth plates (Fig. 6C–E) of the cKO mice, respectively. Type I collagen (COL1) showed lower expression in the trabecular bone of cKO mice than that in the WT mice (Fig. 6F–H). These results indicate that the FAM20B-catalyzed proteoglycans are essential to chondrocyte differentiation and maturation, as well as the subsequent ossification.

### *Fam20B*-deficient chondrocytes exhibited gaihn of function in multiple signaling pathways

**Figure 7:**
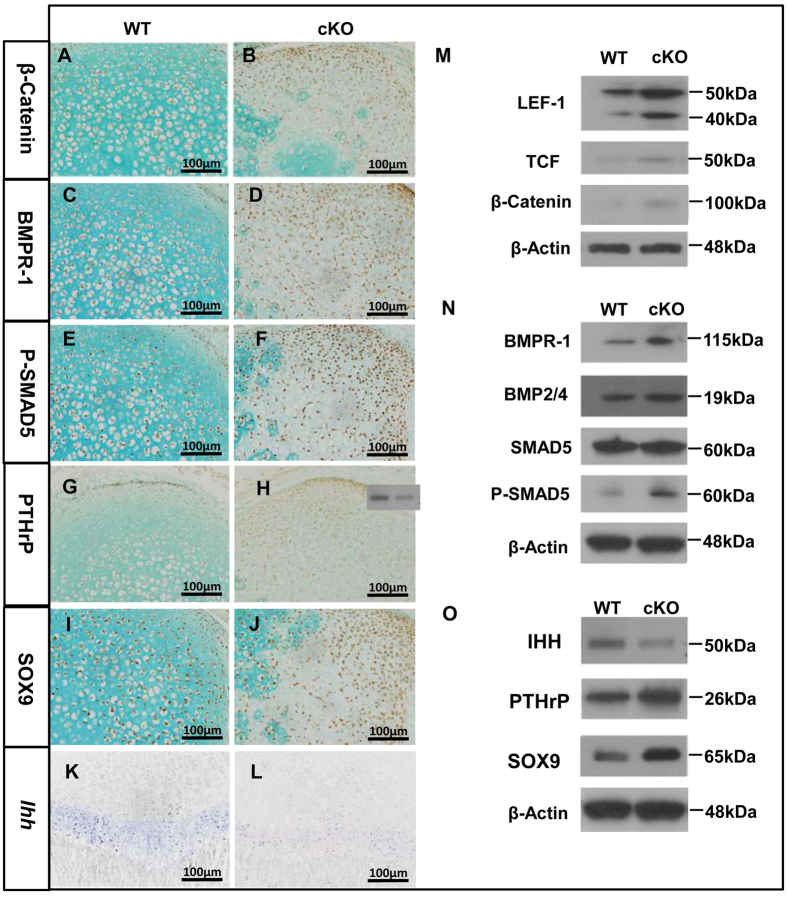
Signaling changes in the epiphyseal cartilage of *Osr2-Cre; Fam20B*^*flox/flox*^ mice. (**A–J)** IHC staining showed a higher expression of β-catenin, BMPR-1A, P-SMAD5, PTHrP, and SOX9 in the epiphyseal cartilage of femurs in the P4 cKO mice than normal. (**K**,**L**) ISH staining of showed a reduced expression of *Ihh* in the prehypertrophic chondrocytes in cKO mice. (**M**) Western immunoblotting confirmed the increased expression of LEF-1,TCF, and β-catenin in the epiphyseal cartilage of cKO mice. (**N**) Western immunoblotting showed an upregulation of BMPR-1A, BMP2/4, SMAD5, and P-SMAD5 BMP in the epiphyseal cartilage of cKO mice. (**O**) Western immunoblotting revealed an upregulation of PTHrP and SOX9 in the epiphyseal cartilage of cKO mice, and a downregulation of IHH in the femur of cKO mice. Gels have been run under the same experimental conditions. The blots of LEF1, TCF, and β-Catenin were cropped from full-length gel as indicated in Figure S. **E,F**; The blots of BMPR-1, BMP2/4, SMAD5, and p-SMAD5 were cropped from full-length gel as indicated in Figure S **H–K**; The blots of IHH, PTHrP, and SOX9 were cropped from full-length gel as indicated in Figure S **M–O; N** = 6 per group. Scale bars, 100 μm.

Using IHC method, we identified upregulation of β-catenin, BMPR-1A, p-SMAD5, PTHrP, and SOX9 in the epiphyseal cartilage of cKO mice (Fig. 7A–J). Canonical WNT and BMP signaling regulate chondrocyte growth and differentiation. Central to the canonical WNT pathway is β-catenin, which activates downstream gene expression through binding to the T-cell factor/lymphoid enhancer factor (TCF/LEF) transcriptional factors. We found overexpression of β–catenin and LEF-1 in the epiphyseal cartilage of cKO mice using Western immunoblotting method (Fig. 7M). In addition, BMP receptor 1A (BMPr-1A), BMP2/4, and p-SMAD1/5 were upregulated in the epiphyseal cartilage (Fig. 7N), suggesting that gain-of-function of BMP signaling was involved in the cartilage defects of *Fam20B-*cKO mice.

IHH and PTHrP coordinate chondrocyte proliferation and maturation through a negative feedback loop. IHH stimulates chondrocyte differentiation and PTHrP transcription; PTHrP in turn suppresses chondrocyte maturation associated with IHH expression^7^. ISH of *Ihh* showed a reduced expression in the prehypertrophic chondrocytes of cKO mice (Fig. 7K,L). Western immunoblotting of IHH, PTHrP, and SOX9 revealed an upregulation of PTHrP and SOX9 in the epiphyseal cartilage of cKO mice, and a downregulation of IHH in the femur of cKO mice (Fig. 7O). These results suggest that GAG deficiency may interfere with the IHH/PTHrP feedback loop. We did not detect significant changes in FGF and TGF-β signaling in the *Osr2-Cre;Fam20B*^*flox/flox*^ mice (data not shown).

### WNT inhibitor partially rescued the postnatal ossification in *Osr2-Cre;Fam20B* ^*flox/flox*^ mice

**Figure 8:**
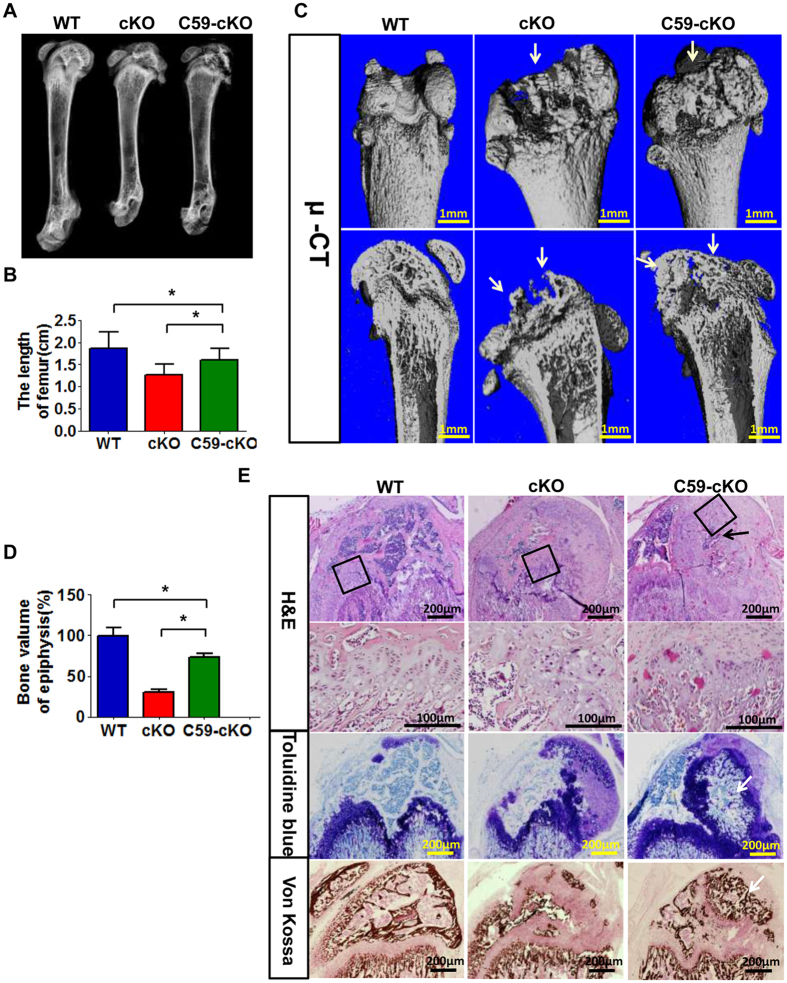
WNT inhibitor partially rescued the postnatal bone elongation and endochondral ossification in *Osr2-Cre;Fam20B*^*flox/flox*^ mice. (**A**,**B**) X ray and quantitation analyses of femurs from 4-week-old mice showed improved bone length of the C59-treated cKO mice. The bone length of C59-treated cKO mice was shorter than WT. n = 6 in B *P < 0.01. (**C**) Micro-CT analysis of femurs from 4-week-old mice. Upper panels: the reconstructed 3D images of femurs showed improved epiphyseal bone formation (arrows) and a better shaped metaphysis in the C59-treated cKO mice than the untreated cKO mice (arrows). Lower panels: the sagittal sections of μ-CT reconstruction showed an improved epiphyseal bone formation in the C59-treated cKO mice (arrows). (**D**) Quantitation of bone volume showed a significant improvement in the epiphyseal areas of C59-treated cKO mice than the untreated cKO mice (n = 6). *P < 0.01. (**E**) H&E, Toluidine blue and *Von Kossa* staining. The cKO mice had poorly differentiated chondrocytes in the popliteal side of the epiphysis compared with the same area in the C59-treated cKO mice (arrows). *p < 0.05. N = 6 per group. ANOVA was used followed by the Bonferroni method of multiple comparisons to determine the difference among groups. Scale bars: 200 μm.

To determine the role of overactivated WNT signaling in the defective chondrocytes and postnatal ossification of the *Osr2-Cre;Fam20B*^*flox/flox*^ mice, we administered WNT inhibitor C59 intraperitoneally to the 5-day-old mice for 2 weeks. X ray and quantitation analyses showed improvement of bone length in the C59-treated cKO mice, which, however, was still shorter than WT (Fig. 8A,B). Micro-CT analysis showed improved epiphyseal bone formation and a better shaped metaphysis in the C59-treated cKO mice (Fig. 8C). Quantitation of bone volume in the epiphyseal areas revealed a significant improvement of the femurs in C59-treated cKO mice than the untreated cKO mice, while the improved bone volume was still significantly lower than that of WT mice (Fig. 8D). H&E, Toluidine blue and *Von Kossa* staining showed poorly differentiated chondrocytes in the popliteal side of the epiphysis in the cKO mice whereas the same areas of C59-treated cKO mice showed initiation of ossification (Fig. 8E). These results suggest that the FAM20B-catalyzed proteoglycans may regulate these processes partially through the mediation of WNT signaling. We also administrated BMP inhibitor LDN-193189 to the *Osr2-Cre;Fam20B*^*flox/flox*^ mice but did not observe any significant improvement in the chondrocyte differentiation and bone formation (data not shown).

## Discussion

In this study, we demonstrated the critical roles of FAM20B-catalyzed proteoglycans in the mediation of a signaling network composed of multiple pathways that control chondrocyte differentiation and proliferation. The skeletogenesis delay in the *Osr2-Cre;Fam20B*^*flox/flox*^ mice is inconsistent with the results from a previous study^18^ in which zebra fish with *Fam20b*/xylosyltransferase1 (*xylt1*) mutations demonstrated an accelerated development and an expanded ontology of genes regulating the rate of skeletogenesis. The discrepancy between these animal models may reflect a diversity of FAM20B function in different species with or without combined gene mutations, or arise from other circumstances under which *Fam20B* was inactivated, i.e., constitutively versus conditionally.

The *Osr2-*Cre displayed differential expression between the popliteal side and the patellar side of epiphyseal cartilage. This unique pattern led to completely different fates between the Cre^+^ and Cre^−^ chondrocytes in *Osr2-Cre;Fam20B*^*flox/flox*^ mice: The Cre^+^ (*Fam20B*-deficient) chondrocytes developed underdifferentiation and overproliferation, and failed to initiate ossification at the popliteal side of the secondary ossification center, while the Cre^−^ (*Fam20B* -intact) chondrocytes underwent nearly normal differentiation and proliferation, and successfully transformed to spongy bones at the patellar side of the secondary ossification center (Figs 2, 3 and 8), suggesting that FAM20B and FAM20B-catalyzed proteoglycans are essential components for chondrocyte differentiation and the formation of secondary ossification center, which likely act in an autonomous manner during these processes.

We further demonstrated that inactivation of *Fam20B* in chondrocytes led to gain of function in multiple signaling pathways. It has been well documented that the membrane-attached and extracellular proteoglycans are able to regulate growth factors and morphogens through binding to them as receptor/co-receptor, or by creating a diffusion gradient^22^. Proteoglycans can either stimulate or inhibit signaling activity, depending on the biological context^23^. During postnatal endochondral ossification, WNT signaling promotes chondrocyte differentiation and the final maturation, and thus plays critical roles in the development of a secondary ossification center^24^,^25^. In the *Fam20B-*deficient chondrocytes, WNT signaling was upregulated, whereas the postnatal endochondral ossification were inhibited, suggesting a different role of WNT signaling in this specific biological context. In fact, the function of the WNT proteins can drastically change temporally or spatially, depending on the stage of cartilage formation and the molecules involved^26^. The partially rescued bone formation in the secondary ossification center in the *Fam20B*-cKO mice via the administration of WNT inhibitor indicates that the overactivated WNT signaling was involved in the defective chondrocyte differentiation and postnatal bone formation.

Given that administering WNT inhibitor to the *Osr2-Cre;Fam20B*^*flox/flox*^ mice did not significantly rescue the chondrosarcoma phenotype, we envision that the chondrosarcoma may be associated with molecular changes of other pathway(s). In addition to the mediation on WNT signaling, proteoglycans (especially heparan sulfate proteoglycans) also regulate hedgehog (HH) signaling^27^. Previous studies have demonstrated that heparan sulfate deficiency in the *Ext1*-knockout mice altered the IHH diffusion gradient, expanded the expression of PTCH1 and PTHrP, and eventually led to osteochondroma in the metaphyseal cartilage^28^,^29^. Although it remains elusive as to whether the chondrosarchoma in *Osr2-Cre;Fam20B*^*flox/flox*^ mice was associated with a gradient disturbance of IHH as in the *Ext1*-knockout mice, the downregulated IHH and the upregulated PTHrP and Sox9 likely contributed to the differentiation defects due to their well-documented functions^30^.

Emerging evidences suggest that proteoglycans have important roles in angiogenesis^31^,^32^ and osteoclastgenesis^33^. Based on the histology of P7 femurs (Fig. 2A,B), *Fam20B*-cKO mice appeared to have a delayed vasculature invasion into the secondary ossification center. Accordingly, TRAP-positive cells (i.e., osteoclasts) in the long bones of cKO mice were less than those in the WT mice (Fig. S2), suggesting that the delayed ossification in cKO mice may be associated with a failure in vasculature invasion and the ensuing osteoclast recruitment. However, the expression of angiogenesis markers, VEGF and CD34, was upregulated in the epiphyseal cartilage of cKO mice (Fig. S3). A possible explanation for these contradictory observations is that the upregulation of angiogenesis markers might be a secondary effect from the overgrowth of chondrosarcoma, while the underdifferentiated chondrocytes could not initiate the vasculature and ossification. Apparently, more investigations are needed in the future to determine the significance of these alterations in the *Fam20B*-cKO mice.

FAM20B-catalyzed xylose phosphorylation is essential to initiating the GAG assembly of both heparin sulfates and chondroitin sulfates^17^. However, several lines of evidence suggest that the phosphorylation of xylose should be under tight control by the biological context and the specificity of substrates (the core proteins of proteoglycans). First, the constitutive inactivation of mouse exostosin glycosyltransferase 1 (EXT1), a polymerase essential to HS assembly, led to embryonic lethality at E6-7.5^34^, while constitutive inactivation of mouse *Fam20B* caused embryonic death at E13.5^19^. The longer survival time of *Fam20B*-knockout mouse embryos suggests that the GAG deficiency arising from *Fam20B*-deficiency may involve a different spectrum of HSPGs or affects fewer amounts of GAGs compared to those in the *Ext1*-knockout mice. Second, we and Eames *et al*.^14^,^18^ demonstrated that GAGs in the *Fam20B*-deficient cartilage were remarkably reduced but not eliminated. Future investigations are warranted to determine the FAM20B substrates and the biological context for xylose phosphorylation.

Taken together, our study demonstrates that the FAM20B-catalyzed proteoglycans are critical orchestrators for multiple signaling pathways in the regulatory network that controls chondrocyte differentiation and postnatal bone formation and are involved in tumorigenesis in cartilage.

## Materials and Methods

### Animals

The *Fam20B*^*flox/flox*^mice were crossbred with *Osr2-Cre* transgenic mice (Jackson Laboratory, ME, USA). The resulting *Osr2-Cre;Fam20B*^*flox/+*^ mice were inbred to get *Osr2-Cre;Fam20B*^*flox/flox*^mice, *i.e., Osr2-Cre*-mediated conditional knockout (cKO) mice. Tail biopsies were analyzed by PCR genotyping with primers specific for *Cre* transgene and *Fam20B-*floxed allele, as previously described^21^. The *ROSA26-Tomato* mice (Jackson laboratory) were bred with *Osr2-Cre* mice to trace the Cre^+^ lineage. All animal procedures were approved by the Institutional Animal Care and Use Committee of Texas A&M-Baylor College of Dentistry (Dallas, TX, USA) and performed in accordance with the National Institutes of Health *Guide for the Care and Use of Laboratory Animals*.

### X-ray radiography and micro-computed tomography

The femurs dissected from 4-, 7-, and 30-day-old *Osr2-Cre;Fam20B*^*flox/flox*^ mice and their WT littermates were analyzed by plain x-ray radiography (Faxitron Bioptics, AZ, USA). The femurs from 30- and 60-day-old mice were analyzed by micro-computed tomography (micro-CT) using a μ-CT35 imaging system (Scanco Medical, PA, USA) at medium resolution (7.0 μm slice increment). The images were reconstructed with EVS Beam software using a global threshold of 200 Hounsfield units, as previously described^35^.

### Alizarin red/alcian blue staining of the skeleton

Seven-day-old *Osr2-Cre;Fam20B*^*flox/flox*^ mice and their WT littermates were skinned, eviscerated and fixed in 95% ethanol. Alizarin red/Alcian blue staining of the skeletons was performed to visualize the skeleton and cartilage, as previously described^35^.

### H&E staining and safranin O staining

The femurs dissected from 4-, 7-, and 30-day-old *Osr2-Cre;Fam20B*^*flox/flox*^ mice and their WT littermates were fixed by 4% paraformaldehyde in 0.1% diethyl pyrocarbonate (DEPC)-treated PBS solution at 4 °C overnight and then decalcified in 15% EDTA (pH 7.4) at 4 °C for 2 to 8 days. The tissues were processed for paraffin embedding, and 6 mm serial sections were prepared for histological analyses. H&E staining and safranin O staining were performed as previously described^36^.

### Immunohistochemistry (IHC) and *in situ* hybridization (ISH)

IHC staining was performed on sections prepared from femurs of 4-day-old mice using a ABC kit and DAB kit (Vector Laboratories, CA, USA) according to the manufacturer’s instructions, as previously described^37^. The primary antibodies used for IHC analysis are summarized in Table S1. Methyl green was used for counterstaining. The intensity of IHC staining and the number of positively stained cells were measured and calculated using ImageJ 1.44 software following the user guide.

ISH was performed on sections prepared from paraffin-embedded samples. The RNA probes for type X collagen and indian hedgehog (*Ihh*) were labeled with digoxigenin (DIG) using a RNA labeling kit (Roche Life Science, IN, USA), according to the manufacturer’s instructions. The DIG-labeled RNA probes were detected by an enzyme-linked immunoassay with anti-DIG-AP antibody (Roche Life Science) and alkaline phosphatase chromogen (BCIP**/**NBT) substrate (Vector Laboratories) to produce a blue/purple color for positive signals.

### *Von Kossa* staining

The undecalcified femurs from 7-day-old mice were fixed and processed for methylmethacrylate embedding. Serial sections (10 μm) were prepared and incubated with 1% silver nitrate solution under a 60-W light bulb for 2 hr. The sections were then rinsed in several changes of distilled water, incubated in 5% sodium thiosulfate to remove the unreacted silver, and counterstained with nuclear fast red.

### Western immunoblotting

The epiphyseal cartilage dissected from femurs of 4-day-old mice was crushed into powder in liquid nitrogen and lysed by RIPA buffer (ThermoFisher Scientific, NY, USA) supplemented with a mixture of protease inhibitors (Roche). The lysates were analyzed by sodium dodecyl sulfate polyacrylamide gel electrophoresis (SDS-PAGE), followed by Western immunoblotting using antibodies listed in Table S2.

### Administration of WNT and BMP inhibitors

To determine the role of upregulated WNT and BMP signaling in the cartilage and bone defects in *Osr2-Cre;Fam20B*^*flox/flox*^ mice, we administrated WNT inhibitor C59 (Cellagen Technology, C7641, CA, USA) and BMP inhibitor LDN-193189 (Stemgent Inc., 04–0074, MA,USA) to the *Fam20B*-cKO mice. C59 (1mg/kg body weight in DMSO) or LDN-193189 (0.5 mg/kg body weight in DMSO) was administrated intraperitoneally to the 5-day-old pups every other day for 14 days. The mice were sacrificed at postnatal 28 days and their femurs, were collected for μ-CT, H&E, toluidine blue and Von Kossa staining analyses.

### Statistical analysis

The data were expressed as mean ± SD. The Student’s t-test was used to compare the means between two groups. For analyses where comparisons were made among more than two groups, an analysis of variance (ANOVA) was used followed by the Bonferroni method of multiple comparisons to determine which groups were significantly different from each other. A value of p < 0.05 was considered statistically significant.

## Additional Information

**How to cite this article**: Ma, P. *et al*. Inactivation of *Fam20B* in Joint Cartilage Leads to Chondrosarcoma and Postnatal Ossification Defects. *Sci. Rep.* **6**, 29814; doi: 10.1038/srep29814 (2016).

## Supplementary Material

Supplementary Information
