# Supplementary material for: Inactivation of Fam20B in Joint Cartilage Leads to Chondrosarcoma and Postnatal Ossification Defects
Source: Sci Rep. 2016 Jul 13;6:29814. doi: 10.1038/srep29814 (PMC4942823; doi:10.1038/srep29814)
Supplement: Supplementary Information [file srep29814-s1.pdf]

Inactivation of *Fam20B* in Joint Cartilage Leads to Chondrosarcoma and Postnatal Ossification Defects

Pan Ma[1, 2], Wenjuan Yan[1], Ye Tian[1], Jingya Wang[1], Jian Q. Feng[1], Chunlin Qin[1], Yi-Shing Lisa Cheng[3], Xiaofang Wang[1]*

[1] Department of Biomedical Sciences and Center for Craniofacial Research and Diagnosis, Texas A&M University Baylor College of Dentistry, Dallas, Texas, United States of America

[2] Department of Oral Implantology, Beijing Stomatological Hospital, Capital Medical University, Beijing, People's Republic of China

[3] Department of Diagnostic Sciences, Texas A&M University Baylor College of Dentistry, 3302 Gaston Ave, Dallas, TX, United States of America

Correspondence and requests for materials should be addressed to XW (email: xwang@bcd.tamhsc.edu)

Table S1. Antibodies used for immunohistochemistry staining

Name	Source	Type	Working concentration	Manufacturer
**anti-PCNA**	mouse	Monoclonal	1:400	Santa Cruz
**anti-Ki67**	Goat	Polyclonal	1:600	Santa Cruz
**anti-BCL-2**	Rabbit	Polyclonal	1:300	Santa Cruz
**anti-COL1**	Goat	Polyclonal	1:50	Santa Cruz
**anti-COL2**	Goat	Polyclonal	1:300	Santa Cruz
**anti-β-Catenin**	Mouse	Monoclonal	1:300	Santa Cruz
**anti-BMPR1A**	Rabbit	Monoclonal	1:300	Cell Signaling
**anti-P-SMAD5**	Rabbit	Monoclonal	1:600	Cell Signaling
**anti-PTHrP**	Rabbit	Polyclonal	1:300	Santa Cruz
**anti-Sox9**	Rabbit	Polyclonal	1:600	Santa Cruz

Table S2. Antibodies used for Western immunoblotting

Name	Source	Type	Working concentration	Manufacturer
**anti- HS**	Rat	Monoclonal	1:500	Santa Cruz
**anti- CS**	Mouse	Monoclonal	1:500	Abcam
**anti-COL2**	Goat	polyclonal	1:600	Santa Cruz
**anti-COL10**	Rabbit	polyclonal	1:1000	Santa Cruz
**anti-LEF-1**	Rabbit	Monoclonal	1:1000	Cell Signaling
**anti-TCF**	Rabbit	Monoclonal	1:500	Cell Signaling
**anti-β-Catenin**	Mouse	Monoclonal	1:400	Santa Cruz
**anti-BMPR1A**	Rabbit	Polyclonal	1:1000	Santa Cruz
**anti-BMP2/4**	Rabbit	Polyclonal	1:1000	Santa Cruz
**anti-SMAD5**	Rabbit	Polyclonal	1:1000	Cell Signaling
**anti-IHH**	Rabbit	Polyclonal	1:400	Santa Cruz
**anti-p-SMAD5**	Rabbit	Monoclonal	1:400	Cell Signaling
**anti-PTHrP**	Rabbit	Polyclonal	1:1000	Santa Cruz
**anti-Sox9**	Rabbit	Polyclonal	1:1000	Santa Cruz
**β-ACTIN**	Mouse	Monoclonal	1:3000	Santa Cruz

Figure S1. The full-length gels of cropped blots in Figures 3, 6, and 7

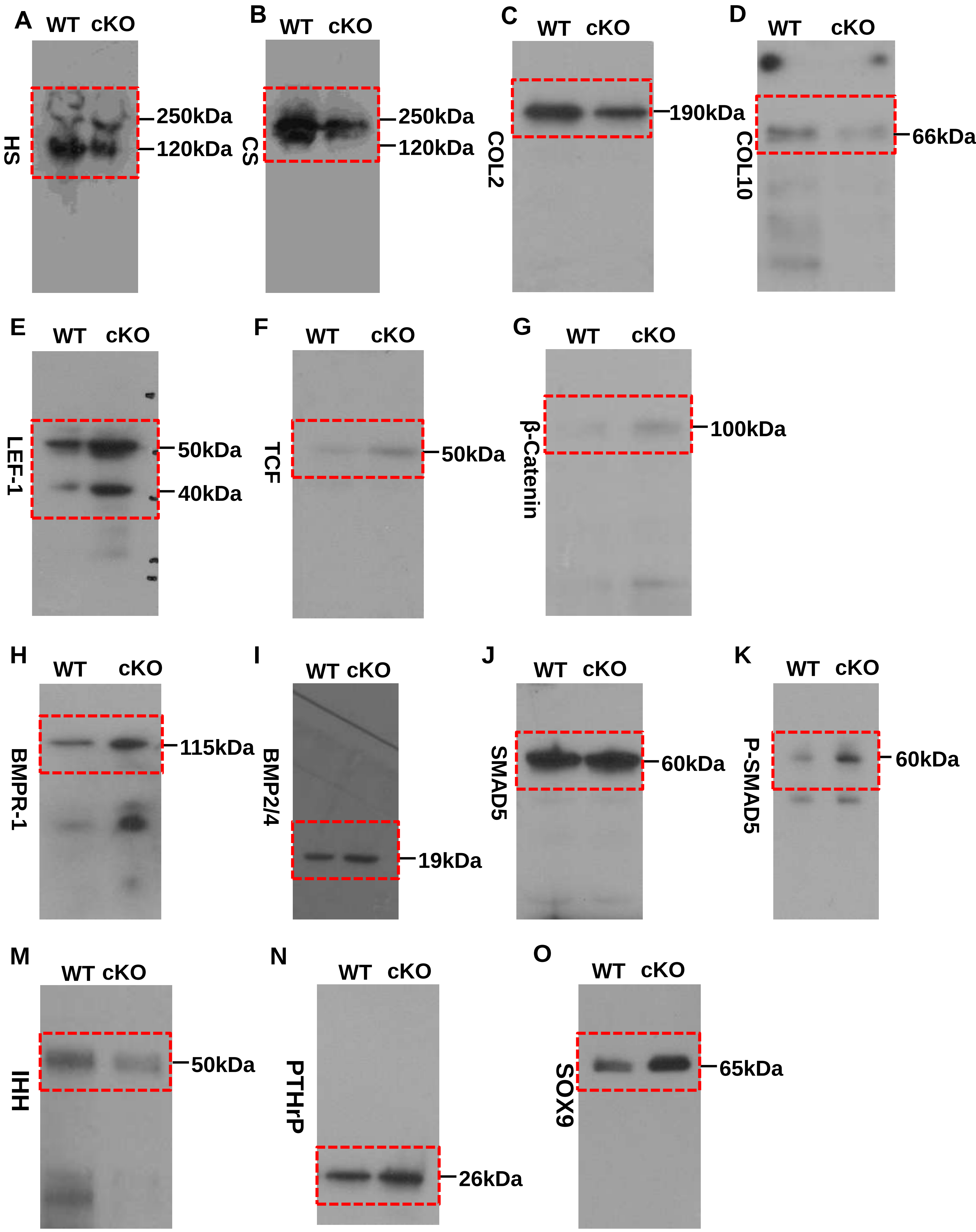

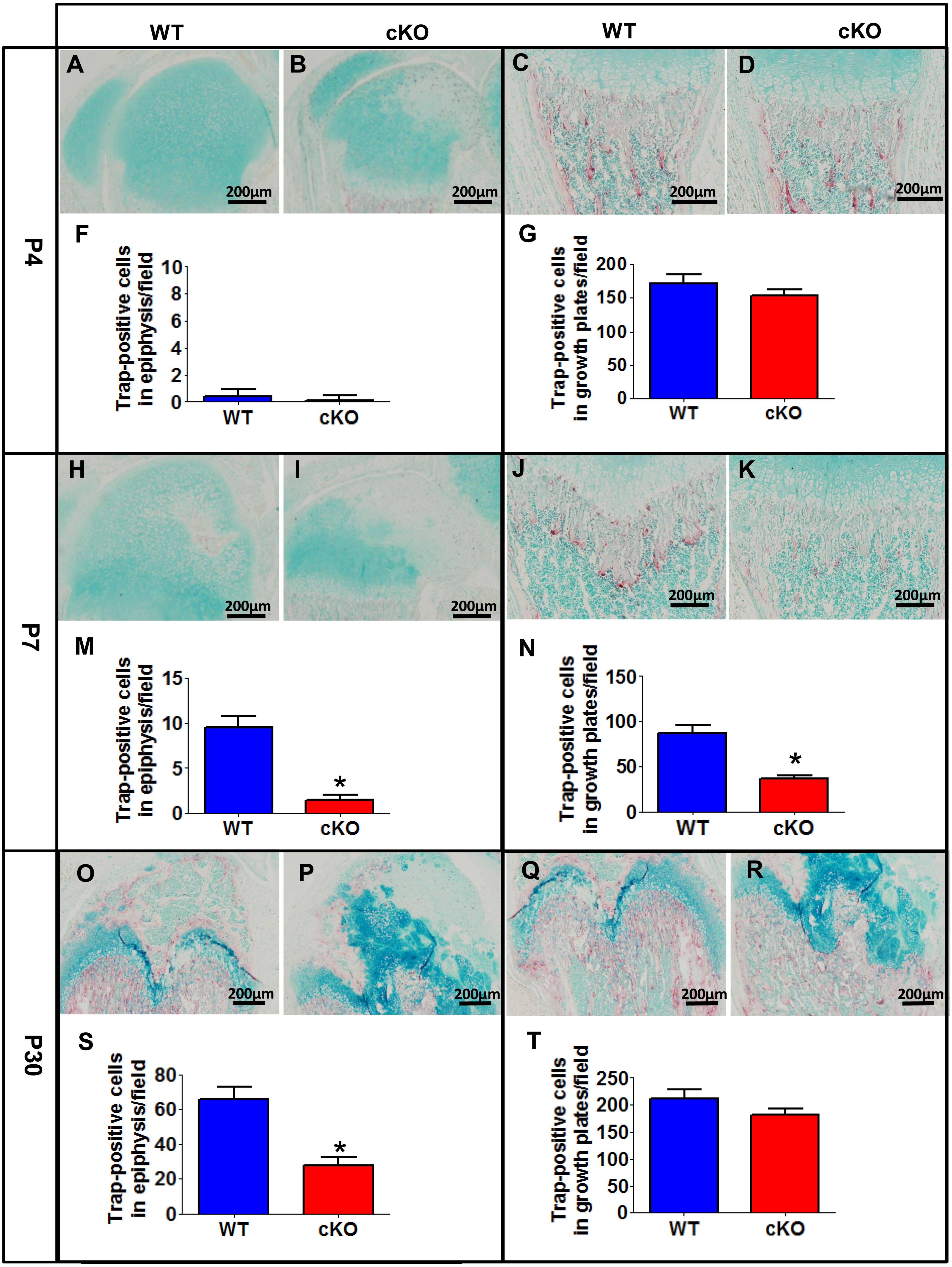

Fig. S2. TRAP staining of femurs in P4, P7, and P30 mice.

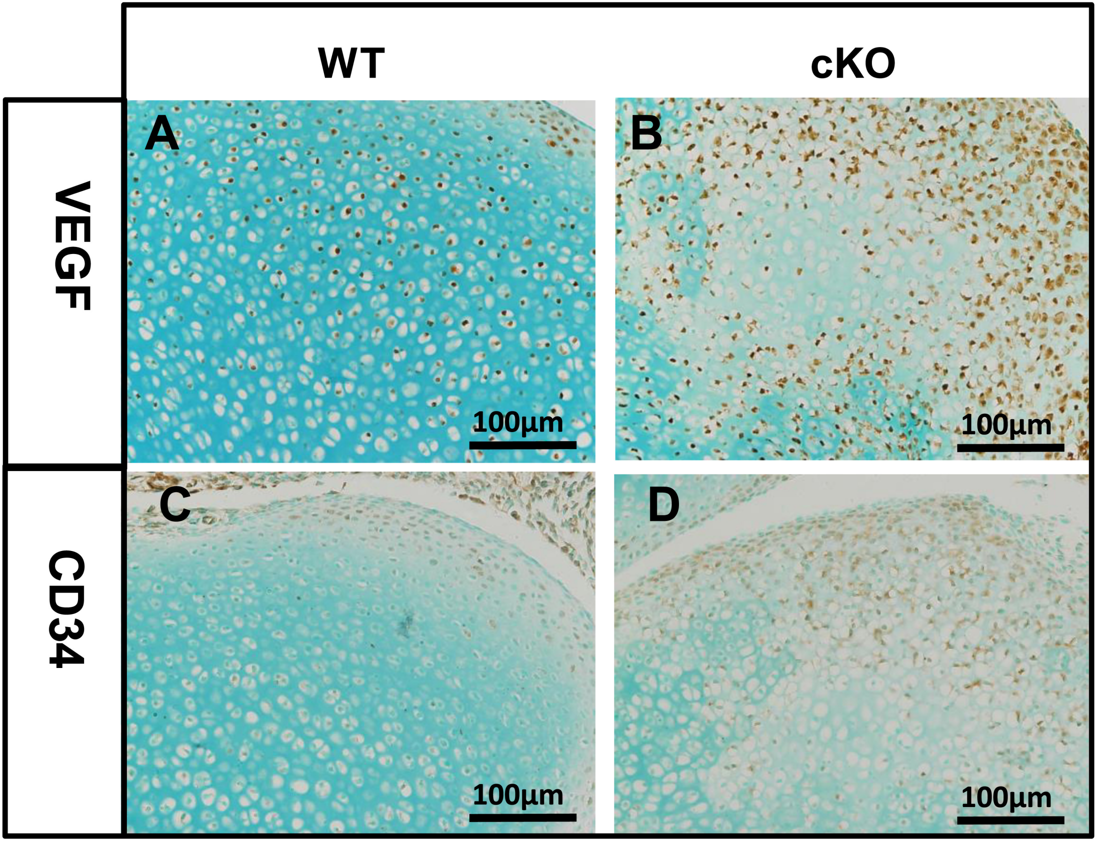

Fig. S3. IHC staining of VEGF and CD34 in P7 mice.
